# Macular Thickness by Age and Gender in Healthy Eyes Using Spectral Domain Optical Coherence Tomography

**DOI:** 10.1371/journal.pone.0037638

**Published:** 2012-05-21

**Authors:** Mehreen Adhi, Sumbul Aziz, Kashif Muhammad, Mohammad I. Adhi

**Affiliations:** 1 Department of Ophthalmology, Dow University of Health Sciences and Civil Hospital, Karachi, Pakistan; 2 New England Eye Center, Tufts Medical Center, Boston, Massachusetts, United States of America; National Eye Institute, United States of America

## Abstract

**Purpose:**

To determine normal macular thickness and its variation by age and gender in healthy eyes using spectral-domain optical coherence tomography (SD-OCT).

**Methods:**

In this cross-sectional analysis, two hundred and twenty eyes of 220 healthy subjects underwent raster scanning using Topcon SD-OCT system, at the Department of Ophthalmology, Dow University of Health Sciences and Civil Hospital Karachi, Pakistan. Macular thickness from all 9 regions of the ETDRS map was documented for each subject. Variations in macular thickness measurements by age and gender were determined.

**Results:**

The 220 subjects had a mean age of 45.3 years (16–80 years). Using the ETDRS map, foveal thickness for all subjects was measured to be 229±20.46 µm. Mean macular thickness for all subjects was 262.8±13.34 µm. Male gender was associated with greater foveal (p<0.0001) and mean macular (p<0.0001) thickness compared to females. There was no association of mean macular thickness (r^2^ = 0.01; p>0.05) and foveal thickness (r^2^ = 0.00004; p>0.05) with age.

**Conclusion:**

We have provided normative data for macular thickness using Topcon SD-OCT system. Our results are comparable to some and vary from other reports using the similar OCT system. Our results suggest that male gender is associated with greater macular thickness, while macular thickness has no association with age in healthy eyes. This is the first normative data for macular thickness from Pakistan; benchmark for diagnosing and monitoring macular pathologies. The values obtained in this study may be useful for comparison with other populations, other SD-OCT systems and future imaging technologies.

## Introduction

Macular edema is a common cause of visual impairment, and the degree of macular thickening is significantly correlated with visual acuity [1]. An increase in retinal thickness due to fluid accumulation is found in many ocular disorders such as diabetic retinopathy, age-related macular degeneration, central serous chorio-retinopathy (CSCR) and retinal vein occlusion. Assessment of macular region is also an important parameter for staging and monitoring of glaucoma [2].

At best, traditional investigations for evaluating macular edema/thickening, such as fundus photography, slit lamp bio-microscopy and fluorescein angiography (FA), can provide only qualitative information, which is relatively insensitive to subtle changes in macular thickness [3]. The introduction of optical coherence tomography (OCT) has revolutionized ophthalmic clinical practice. OCT uses low coherence interferometry of light to examine the retina in vivo [4]. With progression of this technology, a true, non-contact, non-invasive “optical biopsy” of the posterior segment of the eye is achievable. It has enabled clinicians to appreciate refined details of the posterior segment of the eye on a micron scale, and to reliably detect and quantify subtle changes in macular thickness, thus making objective monitoring of disease progression and efficacy of different therapeutic modalities in various ocular diseases plausible [5], [6], [7].

Since its advent, OCT has shown major improvements in technology, with increased resolution of images and higher acquisition speed. Standard OCT systems such as Stratus OCT, uses time-domain detection, achieving scan rates of 400 A-scans per second and an axial resolution of 8–10 µm [5]. More recently, about 7 commercially available Spectral/Fourier domain OCT (SD-OCT) systems provide higher sensitivity, much higher speed of acquisition (greater than 20,000 A-scans per second) and better resolution (5–7 µm), thus making it possible to acquire large, volumetric data sets in a relatively much shorter time frame [5], [8]–[10].

Studies have reported significant differences in macular thickness amongst subjects of different race, gender and age [11]–[14]. These demographic variations may be important parameters when comparing macular thickness measurements and diagnosing ocular diseases. With increasing use of SD-OCT in clinical practice, it is critical to measure macular thickness in healthy eyes as well as to compare these values with the current commercially available OCT systems.

The purpose of this study was to determine the normal macular thickness, and variations in macular thickness by age and gender in healthy eyes using Topcon SD-OCT system.

## Methods

### Subjects

In this cross sectional analysis, two-hundred and twenty eyes of 220 healthy subjects, underwent raster scanning at Department of Ophthalmology, Dow University of Health Sciences, Civil Hospital Karachi, Pakistan, between July 2009 to August 2010. None of the subjects had any previous retinal or choroidal pathology or history of any previous ocular intervention. All subjects had a best-corrected visual acuity of 20/20 or greater and underwent complete ophthalmological examination, including dilated fundus examination. Subjects with myopic refractive error of greater than 5.0 diopters were excluded. The ethical committee of Dow University of Health Sciences approved this study. Verbal informed consent was obtained from all subjects before acquisition of scans. An information sheet approved by the ethics committee was used to describe the purpose of the study to the participants, and to obtain their consent, which was then signed and dated by the researcher obtaining the consent as well as a witness. The ethical committee approved this method since acquisition of OCT images is a non-contact, non-invasive procedure. This study was conducted according to the tenets of Declaration of Helsinki.

### Optical Coherence Tomography (OCT)

OCT scanning was performed using Topcon SD-OCT (3D OCT*-*1000, Mark II*;* Topcon Corporation, Tokyo, Japan). This OCT system has a resolution of 6 µm. As a light source, it uses super luminescent diodes with a wavelength of 840 nm. High speed scanning reduces eye movements and thus, eliminates chances of artifacts. Mobile internal digital fixation patterns allow for varying patient fixation patterns.

One eye per subject was randomly selected for scanning. After pharmacological pupillary dilation and instillation of artificial tears, imaging was performed 3 times in each subject, on the same day, by one experienced operator trained in using the Topcon OCT system. All scans had an image quality factor of 60/100 or greater and were taken as close to the fovea as possible, such that the thinnest point of the macula was imaged, so as to avoid errors in the thickness measurements due to slight differences in positioning. Following acquisition, two independent observers examined the images obtained from each subject for any obvious segmentation errors, which if present, disqualified the image from acquisition in the study and scanning was repeated. If deemed necessary, the Early Treatment Diabetic Retinopathy Study (ETDRS) grid was shifted to compensate for any fixation errors. In addition, the images were deemed acceptable only if the full extent and depth of the retina was clearly distinguishable, and there were no blinking artifacts or eye movements during image acquisition.

### Macular Thickness Measurements

**Figure 1:**
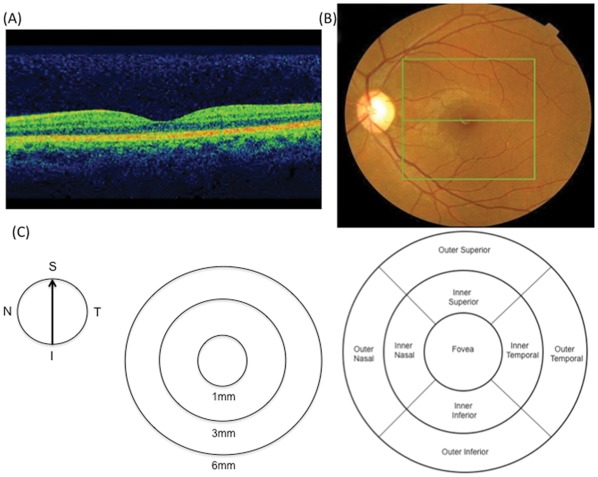
Example of macular thickness measurements obtained with Topcon SD-OCT system. Representative OCT image from a healthy subject (A). Fundus photograph of a healthy subject; the box indicates 6×6 mm scanning area using 3D macular protocol (B). Depiction of standard ETDRS map (C), showing map diameters centered on fovea (left) and 9 standard ETDRS regions (right). I, inferior; N, nasal; S, superior; T, temporal; RPE, retinal pigment epithelium; ETDRS, Early Treatment Diabetic Retinopathy Study; OCT, optical coherence tomography.

The 3D macula protocol was used for macular thickness measurements. It consists of a raster-scan composed of 256×256 (vertical×horizontal) axial scans covering an area of 6×6 mm in the macular region. It reconstructs a false-color topographic image displayed with numeric averages of thickness measurements for each of the 9 map regions within a 6×6 mm area centered on the fovea, as defined by the ETDRS [15]. According to ETDRS map, macula is divided into 9 regions with 3 concentric rings measuring 1 mm (innermost ring), 3 mm (inner ring) and 6 mm in diameter (outer ring) centered on the fovea. The innermost 1 mm ring is the fovea while the 3 mm inner ring and 6 mm outer ring are further divided into four equal regions [Figure 1]. It identifies the layers of the retina and determines macular thickness by measuring the distance between the inner limiting membrane (ILM) and the inner boundary of retinal pigment epithelium (RPE) in each of the 9 regions.

Macular thickness measurements generated by the OCT system in all the 9 regions of ETDRS map were documented from the three scans obtained from each subject, and were averaged for the purpose of analysis. Foveal thickness was defined as macular thickness within the innermost 1 mm ring. Mean macular thickness was defined as the average macular thickness from all 9 regions of ETDRS map.

### Statistical Analysis

Data is presented as mean ± standard deviation. Mann Whitney test was used to determine variations in thickness measurements by gender. Linear regression analysis was used to determine an association of mean macular thickness and foveal thickness with age. Multi-variant analysis with age and gender as independent variables was also performed to determine the variations in thickness measurements by gender when controlled for age, and the associations of age with mean macular and foveal thickness, when controlled for gender. A 95% confidence interval and a 5% level of significance were adopted; therefore, results with a *p*-value less than or equal to 0.05 were considered significant. All descriptive statistics were performed using Graph Pad Prism 5.0 software for Macintosh (Graph Pad Software, La Jolla, CA), except for multi-variant analysis, for which SPSS software for Windows (Version 19.0, SPSS, Chicago, IL) was used.

## Results

The 220 subjects had a mean age of 45.3 years (range 16–80 years). One hundred and thirty two subjects (60%) were males while 88 subjects (40%) were females. The mean ages of males and females were 45.9 years and 44.4 years respectively (p = 0.4). In addition, the mean myopic refractive error in males and females were 1.35 and 1.65 respectively (p>0.05).

Using the ETDRS map, foveal thickness for all subjects was measured to be 229±20.46 µm. The mean macular thickness for all subjects was 262.8±13.34 µm. Macular thickness for all subjects in each of the 9 regions of the ETDRS map is presented in Table 1. Macular thickness was thinnest at the fovea (innermost 1 mm ring), thickest within the inner 3 mm ring and diminished peripherally. It was found to be thickest nasally and thinnest temporally. Overall, the superior and nasal quadrants were thicker than the inferior and temporal quadrants in both the inner 3 mm and outer 6 mm ring.

**Table 1:** Macular thickness measurements by ETDRS* region in 220 healthy eyes using Topcon SD-OCT system.

MACULAR REGION	MACULAR THICKNESS IN 220 HEALTHY EYES (MEAN ± SD)
**Fovea (innermost 1** **mm ring)**	229±20.46 µm
**Inner 3** **mm ring**	
Superior	290.3±18.31 µm
Inferior	287.1±15.46 µm
Nasal	292.6±17.79 µm
Temporal	275.2±22.90 µm
**Outer 6** **mm ring**	
Superior	247±13.63 µm
Inferior	243.2±13.85 µm
Nasal	268.5±15.59 µm
Temporal	232.5±16.51 µm
*Mean Macular Thickness (average from all 9 regions of ETDRS* * *map)*	262.8±13.34 µm

* *ETDRS – Early Treatment Diabetic Retinopathy Study* [*15*] *.*

Male gender was associated with a greater macular thickness in all 9 regions of the ETDRS map compared to females. Foveal thickness in males was measured to be 232.68±21.07 µm, while in females it was 222.87±18.72 µm (p<0.0001). Mean macular thickness in males was 266±14.20 µm, while in females it was 258.21±10.03 µm (p<0.0001). When adjusted for age, males were found to have an increase in mean macular and foveal thickness (p = 0.005 and p = 0.0008 respectively) when compared to females. Macular thickness for both genders in each of the 9 regions of ETDRS map is presented in Table 2.

**Table 2:** Macular thickness measurements in each ETDRS* region by gender in 220 healthy eyes using Topcon SD-OCT system.

MACULAR REGION	MACULAR THICKNESS IN 220 HEALTHY EYES (MEAN ± SD)		
	Males (n = 132)	Females (n = 88)	p value for gender difference
**Fovea (innermost 1** **mm ring)**	232.68±21.07 µm	222.87±18.72 µm	<0.0001
**Inner 3** **mm ring**			
Superior	294.31±20.74 µm	284.88±12.03 µm	0.0002
Inferior	290.85±16.48 µm	282.05±10.85 µm	<0.0001
Nasal	296.88±18.67 µm	286.52±13.61 µm	<0.0001
Temporal	278.55±26.65 µm	270.08±16.12 µm	0.0002
**Outer 6** **mm ring**			
Superior	249.40±15.04 µm	243.85±10.48 µm	0.003
Inferior	245.46±14.69 µm	239.49±11.43 µm	0.02
Nasal	271.71±16.03 µm	264.67±12.87 µm	0.005
Temporal	234.26±18.90 µm	229.58±12.63 µm	0.0007
*Mean Macular Thickness (average from all* *9 regions of ETDRS* * *map*	266±14.20 µm	258.21±10.03 µm	<0.0001

* *ETDRS – Early Treatment Diabetic Retinopathy Study* [*15*] *.*

**Figure 2:**
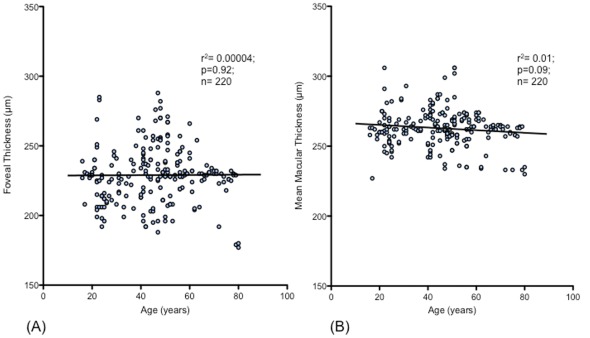
Regression plots of foveal thickness and mean macular thickness vs. age. There is no association of foveal thickness (A) [r^2^ = 0.00004; p = 0.92] and mean macular thickness (B) [r^2^ = 0.01; p = 0.09] with age.

By using linear regression analysis, there was no association of mean macular thickness (r^2^ = 0.01; p>0.05) and foveal thickness (r^2^ = 0.00004; p>0.05) with age [Figure 2]. This was also true when adjusted for gender (p>0.05 and p>0.05 respectively).

## Discussion

OCT has emerged as a useful imaging modality by providing new high-resolution three-dimensional anatomic information about various features of macular pathology [4], [16], and allows clinicians to quantitatively measure macular thickness in a precise, reliable and highly reproducible manner [17], [18].

Of the commercially available OCT systems, a documented variability in macular thickness measurements has been reported [19]–[23], While Stratus OCT selects the inner segment/outer segment (IS/OS) junction as the outer retinal boundary for macular thickness measurements [5], [19]–[22], [24], [25], spectral domain OCT systems select RPE as the outer retinal boundary for thickness measurements, thus leading to an increase in macular thickness reported with these systems, when compared to the TD-OCT systems, while also a slight variability amongst the different SD-OCT systems based on the various scanning protocols and differences in the segmentation algorithms [5], [19]–[23], [25]. Therefore, macular thickness measurements using different OCT systems are not interchangeable [5], [19]–[23], [25]. We used the Topcon SD-OCT, which uses the inner border of RPE as the outer retinal boundary for macular thickness measurements.

Our results show a mean macular thickness of 262.80±13.342 µm and foveal thickness of 229.01±20.464 µm. Giani et al [19] recently reported foveal thickness of 229±24 µm, while Sull AC et al [5] reported a foveal thickness of 231±16 µm in healthy subjects using Topcon OCT system. These values are comparable to our results. However, Hyang et al [22] reported foveal thickness of 221.76±15.95, and Bruce et al [26] reported foveal thickness of 244.83±17.84 µm in healthy subjects using Topcon OCT, which varied significantly from our results. Nevertheless, macular thickness in our subjects decreased from the center towards the periphery of the retina, and was found to be thickest nasally and thinned out temporally. This was consistent with findings reported elsewhere [5], [22].

Demographic variations in macular thickness have been documented previously [11]–[14]. Kashani et al [13] reported mean foveal thickness of 181.0±3.7 µm in African Americans and 200.27±2.7 µm in Caucasians using Stratus OCT. Asefzadeh et al [27] found an overall trend towards a thinner retina in blacks compared to whites using Stratus OCT. Oshitari et al [28] reported a thicker retina in Japanese population in comparison to the US population using Stratus OCT, while Tewari HK et al [29] reported mean foveal thickness in healthy Indian subjects to be 149.16±21.15 µm using Stratus OCT, which was significantly lower than other populations. Grover et al [30] found a significant difference in mean foveal thickness between blacks and whites using Spectralis SD-OCT. When compared to Caucasian and Hispanic subjects, African-American race has been shown to be a predictor of decreased mean foveal thickness and male sex to be a significant predictor of increased mean foveal thickness 13,31. A decrease in macular thickness with age has also been reported 5. Other reports however, have shown no association of macular thickness with age and/or gender [5], [7], [30], suggesting that studies comparing macular thickness measurements should carefully control for age-based, race-based, and gender-based variations 13. Our results showed no association of macular thickness with age, but we found male gender to be associated with greater foveal and mean macular thickness. Thus, demographic variations besides the type of OCT system in use may be important parameters when comparing macular thickness measurements, and diagnosing and monitoring macular pathologies.

Measurement reproducibility is an essential parameter when determining clinical usefulness of an OCT system, particularly when monitoring pathologies. Studies using Topcon OCT system have reported good reproducibility of the system for measuring macular thickness in normal and pathologic states (5,21,22,23,26). As with other SD-OCT systems, reproducibility is better with Topcon OCT system, than with the conventional time-domain systems due to a rapid speed of scan acquisition. We obtained three OCT images from each subject as close to the fovea as possible, excluded images with obvious segmentation errors and adjusted for poor fixation if deemed necessary, with the understanding that slight differences in positioning, eye movements, blinking artifacts and poor fixation may affect the reliability of the macular thickness measurements.

In conclusion, we have provided normative data for macular thickness using the Topcon SD-OCT system. Macular thickness measurements obtained in this study are comparable to some and vary from other reports using the similar OCT system. We have shown that male gender is associated with greater macular thickness, while macular thickness has no association with age in healthy eyes. This study also provides the first normative data for macular thickness from Pakistan; benchmark for diagnosing and monitoring macular pathologies. The values obtained in this study may be useful for comparison with other populations, other SD-OCT systems and future imaging technologies.
